# Cyclic tensile strain facilitates ossification of the cervical posterior longitudinal ligament via increased Indian hedgehog signaling

**DOI:** 10.1038/s41598-020-64304-w

**Published:** 2020-04-29

**Authors:** Daisuke Sugita, Hideaki Nakajima, Yasuo Kokubo, Naoto Takeura, Takafumi Yayama, Akihiko Matsumine

**Affiliations:** 1 Department of Orthopaedics and Rehabilitation Medicine, Faculty of Medical Sciences University of Fukui, 23-3 Matsuoka Shimoaizuki, Eiheiji-cho, Yoshida-gun, Fukui, 910-1193 Japan; 2 Department of Orthopaedic Surgery, Shiga University of Medical Science, Setatsukiwachou, Otsu Shiga, 520-2192 Japan

**Keywords:** Mechanisms of disease, Neurodegeneration

## Abstract

The pathomechanisms of initiation and progression of ossification of the posterior longitudinal ligament (OPLL) are unclear. Indian hedgehog (Ihh) and related signaling molecules are key factors in normal enchondral ossification. The purpose of this study is to investigate the contribution of mechanical strain to OPLL and the relationship of Ihh with OPLL. Sections of the posterior longitudinal ligament (PLL) were obtained from 49 patients with OPLL and from 7 patients without OPLL. Cultured PLL cells were subjected to 24 hours of cyclic tensile strain. To identify differentially expressed genes associated with cyclic tensile strain, microarray analysis was performed. Kyoto Encyclopedia of Genes and Genomes (KEGG) analysis identified upregulation of various genes, particularly of the Hedgehog signaling pathway; *Ihh* and related genes had increased expression compared with controls after 24-hour cyclic tensile strain. In immunoblotting analysis, Ihh, Runx2, Sox9, Gli2, Gli3, and smoothened (SMO) had significantly increased expression after 6- or 12-hour cyclic tensile strain. OPLL samples were strongly immunopositive for Ihh, Sox9, Runx2, Gli2, Gli3, and SMO in the ossification front of OPLL. These results suggest that cyclic tensile strain induces abnormal activation of Ihh and related signaling molecules, and this might be important in the ossification process in OPLL.

## Introduction

Ossification of the posterior longitudinal ligament (OPLL) is characterized by pathological bone formation and has been extensively investigated using various approaches^1–3^. Ossification commences in the vertebral posterior longitudinal ligaments, with a particular predilection for the cervical area, but intensifies and spreads with progression of the disease, resulting in osseous projections and compression of the spinal cord^4^. Myelopathy symptoms due to OPLL are common in middle-aged patients, especially around age 50 years. This pathological entity is common in northeast Asians, and involvement of certain genes was identified in a recent genetic analysis^5–7^. Histologically, the ossifying extension process in OPLL resembles that of enchondral ossification in the growth plates of long bones^8,9^. Our group reported that the ossification process in OPLL and ossification of the ligamentum flavum (OLF) involve chondrocyte differentiation with unique expression of various transcriptional factors. Furthermore, we speculated that differentiation of hypertrophic chondrocytes around the calcified area of the ossification front was closely related to the ossification process^10,11^.

During enchondral ossification of the growth cartilage, Indian hedgehog (Ihh) signaling plays an important role in differentiation and maturation of chondrocytes^12,13^. In our previous study, we suggested that Ihh signaling might also be important in the ossification process in OPLL, as well as in enchondral ossification. We found that expression levels for Ihh, parathyroid hormone-related peptide (PTHrP), and Sox9 were higher in cultured ligament cells derived from the posterior longitudinal ligament (PLL) of patients with OPLL than in those from the PLL of patients without OPLL. In addition, in an immunohistochemical study, we observed expression of Ihh in the ossification front in patients with OPLL^14^.

Ossifying extension in OPLL may be influenced by genetics, endocrine disorders, and other local factors, including cyclic tensile strain^15–17^. The posterior longitudinal ligament is a two-layer structure that is subjected to distraction stress longitudinally and regulates spinal instability. The superficial layer is in close contact with the dura mater and bridges 3 or 4 vertebra, whereas the deep layer is posterior to the vertebral body and connects two adjacent vertebra. Due to these anatomical features, the PLL has distraction strain along its longitudinal axis, and a large mechanical overload. This mechanical stress is important in the ossification process. Previously, we reported that cultured cells derived from ossified plaques of patients with OLF had upregulated mRNA expression for factors involved in the Wnt/β-catenin signaling pathway after 24-hour cyclic tensile strain^18^. In a recent clinical study in patients with cervical OPLL, cervical laminoplasty with instrumented fusion suppressed progression of OPLL in comparison with stand-alone laminoplasty^19,20^. In this study, we used microarray analysis to identify activated signaling pathways in association with cyclic tensile strain. We focused on Ihh and genes in the Ihh signaling pathway, which are important in enchondral ossification of limbs, to determine if these factors are affected by cyclic tensile strain. Thus, the aim of the study is to investigate the effect of cyclic tensile strain on expression of Ihh and related signaling factors in cultured OPLL cells and to evaluate their localization in the ossification front immunohistochemically, with the goal of defining the relationship between Ihh and OPLL.

## Results

A summary of demographic data of the patients in the study is given in Table 1.

**Table 1:** Demographic data for patients in the study.

	Case	Type of OPLL	Age/Sex	Most affected segment	Used for	
OPLL	1	mixed	59/M	C5–6	cell culture	microarray immunoblotting
	2	continuous	68/M	C4–5		microarray immunoblotting
	3	mixed	60/M	C5–6		microarray
	4	mixed	71/M	C5–6		immunoblotting
	5	mixed	75/M	C5–6		immunoblotting
	6	mixed	68/M	C6–7		immunoblotting
	7	mixed	63/F	C5–6		immunoblotting
	8	segmental	79/M	C4–5		immunoblotting
	9	mixed	73/F	C3–4		immunoblotting
	10–49	mixed (n = 12) continuous (n = 5) segmental (n = 23)	Ave. 69.9/ M (n = 23) F (n = 17)	C3–4 (n = 6), C4–5 (n = 11), C5–6 (n = 18), C6–7 (n = 5)		immunohistochemistry
	**Case**	**Diagnosis**	**Age/Sex**	**Affected disc level**	**Used for**	
Non-OPLL	1	CDH	56/M	C4–5	cell culture w/ or w/o immunohistochemistry	microarray immunoblotting
	2	CDH	60/M	C5–6		microarray
	3	CDH	71/M	C5–6		immunoblotting
	4	CDH	63/F	C4–5		immunoblotting immunohistochemistry
	5	CDH	75/F	C4–5		immunoblotting
	6	CDH	61/F	C5–6		immunoblotting immunohistochemistry
	7	CDH	58/M	C5–6		immunoblotting immunohistochemistry

PLL: posterior longitudinal ligament.

OPLL: ossification of the posterior longitudinal ligament.

CDH: cervical disc herniation.

### Cultured cell ALP activity and morphology/viability

**Figure 1:**
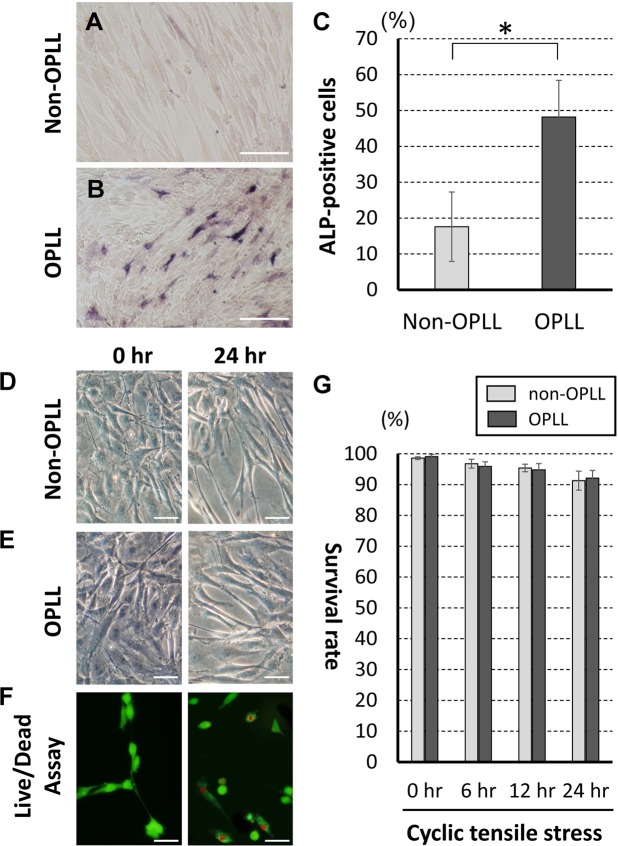
ALP activity in non-stressed cultured spinal ligament cells. (**A**) non-OPLL cells. (**B)** OPLL cells. (**C**) Rates of ALP-positive cells (n = 3 each). *p < 0.05. Cultured spinal ligament cells from non-OPLL (**D**) or OPLL (**E**) patients exhibited a fibroblast-like, spindle-shaped appearance. (**F**) Green cells are live and red cells are dead in the Live and Dead assay. Scale bar = 50 μm. (**G**) Cyclic tensile strain for 24 hours did not change the morphological findings or the viability of the cultured cells (n = 3 each).

To assess osteogenic differentiation, OPLL and non-OPLL cultured cells were stained ALP (red-purple stained cells) (Fig. 1A,B). Under non-stressed conditions, the ALP-positive rates were 48.2 ± 10.2% in OPLL cells and 17.6 ± 9.7% in non-OPLL cells (Fig. 1C). Morphologically, the cultured posterior longitudinal ligament cells exhibited a fibroblast-like, spindle-shaped appearance. The shapes of the cultured cells were almost the same in non-OPLL and OPLL samples (Fig. 1D,E). Under cyclic tensile strain, the viabilities of the attached non-OPLL and OPLL cells were 91.3 ± 3.08% and 92.1% ± 2.53%, respectively, in a 24-hour strain. At each time point, the viability of stressed OPLL cells did not differ significantly from that of non-OPLL cells (Fig. 1F,G).

### Identification of upregulated genes using Gene Ontology (GO) and Kyoto Encyclopedia of Genes and Genomes (KEGG) analysis

Microarray analysis was performed to identify differentially expressed genes associated with OPLL after cyclic tensile strain. The significant upregulated genes in OPLL cultured cells compared to those in non-OPLL cells (difference of intensity >2.0) under non-stress condition and 24-hour cyclic tensile strain were identified by all GO terms in the biological process (see Supplementary Table S1 online) and all KEGG terms (Table 2). Based on the results of the KEGG analysis, we found 7 candidate genes (Ihh, SMO, PTCH1, PTCH2, Gli2, Gli3, and STK36) from the Hedgehog signaling pathway among 27 genes that were significantly upregulated under 24-hour cyclic tensile strain. Changes in expression levels of Ihh and related genes in OPLL and non-OPLL cells shown as a ratio (24 hours/0 hour) are summarized in Table 3. Expression of Ihh, Runx2, Sox6, and vascular endothelin growth factor (VEGF) increased by >50% after cyclic tensile strain in all OPLL samples (case 1–3). Other genes with increased expression of >50% included Sox9, COL11A2, COL6A1, and bone morphogenetic protein (BMP2) in OPLL sample case 1, PTHrP in OPLL sample case 2, and COL11A2, patched 1 (PTCH1), Gli3, and serine/threonine protein kinase 36 (STK36) in OPLL sample case 3. In contrast, only Sox9 expression increased by >50% in non-OPLL sample case 1.

**Table 2:** High frequency pathways identified by KEGG/pathway analysis for genes in OPLL cultured cells with expression changes >2.0-fold compared to those in non-OPLL cultured cells.

Rank	Pathway	count	p-value
**After 24-hour cyclic tensile strain**			
1	Influenza A	22	0.0000043
2	ECM-receptor interaction	12	0.00058
3	Herpes simplex infection	18	0.00087
4	Cell adhesion molecules (CAMs)	15	0.0014
5	Rheumatoid arthritis	11	0.0023
6	Focal adhesion	18	0.0031
7	Rap1 signaling pathway	18	0.0038
8	Inflammatory mediator regulation of TRP channels	11	0.0051
8	Measles	13	0.0062
9	Pathways in cancer	27	0.0065
10	Hedgehog signaling pathway	7	0.0071
11	Glycerolipid metabolism	8	0.0075
12	Malaria	7	0.012
13	Thyroid hormone signaling pathway	11	0.015
14	Phagosome	13	0.015
15	Hepatitis C	12	0.016
16	Arrhythmogenic right ventricular cardiomyopathy (ARVC)	8	0.016
17	Epithelial cell signaling in Helicobacter pylori infection	8	0.016
18	Dilated cardiomyopathy	9	0.017
19	Natural killer cell mediated cytotoxicity	11	0.022
20	Toll-like receptor signaling pathway	10	0.024
21	Hepatitis B	12	0.028
22	ABC transporters	6	0.029
23	Long-term depression	7	0.030
24	Transcriptional misregulation in cancer	13	0.032
25	beta-Alanine metabolism	5	0.033
26	Hypertrophic cardiomyopathy (HCM)	8	0.034
27	Oxytocin signaling pathway	12	0.035
28	Leukocyte transendothelial migration	10	0.038
29	Ovarian steroidogenesis	6	0.043
30	Fc gamma R-mediated phagocytosis	8	0.048
**Under non-stress condition**			
1	Systemic lupus erythematosus	22	0.000000036
2	Alcoholism	23	0.0000011
3	Viral carcinogenesis	18	0.0025
4	Hypertrophic cardiomyopathy (HCM)	10	0.0030
5	Cell adhesion molecules (CAMs)	14	0.0034
6	ECM-receptor interaction	10	0.0063
7	Inflammatory mediator regulation of TRP channels	10	0.013
8	Arrhythmogenic right ventricular cardiomyopathy (ARVC)	8	0.015
9	Dilated cardiomyopathy	9	0.016
10	Regulation of actin cytoskeleton	16	0.016
11	Influenza A	14	0.017
12	Legionellosis	7	0.018
13	MAPK signaling pathway	18	0.019
14	Oxytocin signaling pathway	12	0.031
15	Measles	11	0.034
16	Estrogen signaling pathway	9	0.038
17	p53 signaling pathway	7	0.045

**Table 3:** Genes related to Hedgehog signaling pathway analyzed by microarray.

Gene	Accession number	OPLL			Non-OPLL	
		Ratio^a^ (case 1)	Ratio^a^ (case 2)	Ratio^a^ (case 3)	Ratio^a^ (case 1)	Ratio^a^ (case 2)
Indian hedgehog (Ihh)	NM_002181	2.68	1.72	2.05	1.05	0.90
Sox9	NM_00346	10.81	1.18	1.28	1.96	1.35
Runt-related gene2 (Runx2)	NM_004348	3.98	1.54	1.85	1.13	1.05
Collagen type 11A2 (COL11A2)	NM_080680	3.91	1.20	1.75	0.62	0.75
Collagen type 6a1 (COL6A1)	NM_001848	1.78	1.15	1.29	1.03	0.90
Parathyroid hormone (PTH)	NM_000315	1.02	1.77	1.21	0.44	0.52
Smoothened (SMO)	NM_005631	0.86	1.08	1.29	0.76	0.81
Patched 1 (PTCH1)	NM_001083602	0.74	1.38	1.75	0.63	0.77
Patched 2 (PTCH2)	NM_003738	1.16	0.81	1.31	0.73	0.90
Sox5	NM_152989	0.89	1.42	1.36	0.91	0.85
Sox6	NM_017508	10.12	2.52	3.49	0.89	0.80
Gli2	NM_005270	1.08	0.62	0.83	0.57	0.71
Gli3	NM_00168	1.20	1.48	2.24	0.85	0.80
Vascular endothelial growth factor (VEGF)	NM_001025366	1.95	2.97	1.55	0.99	1.10
Smad3	NM_005902	1.06	0.67	0.51	0.38	0.49
Bone morphogenetic protein2 (BMP2)	NM_001200	2.00	0.59	1.17	0.55	0.65
Bone morphogenetic protein 4 (BMP4)	NM_001202	0.75	1.31	0.94	0.21	0.35
Fibroblast growth factor receptor3 (FGFR3)	NM_00142	1.22	1.15	1.51	0.29	0.43
Signal transducers and activator of transcription (STAT1)	NM_139266	0.62	1.18	1.08	0.87	0.82
Serine /threonine protein kinase 36 (STK36)	NM_015690	1.36	1.26	1.74	0.71	0.93

Ratio^a^: the change in expression of each gene is shown as a ratio (24 hours/0 hour).

### Immunoblot analysis

**Figure 2:**
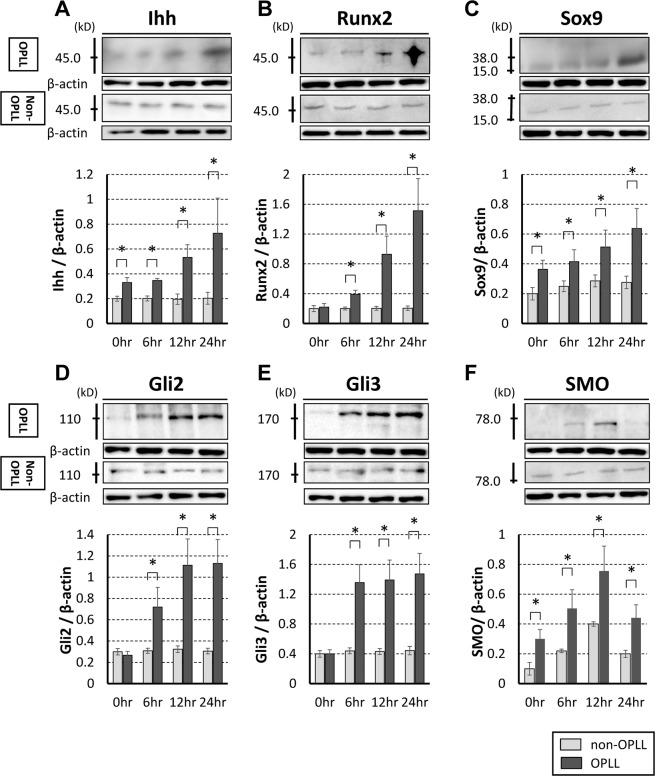
Immunoblotting analysis of Ihh (**A**), Runx2 (**B**), Sox9 (**C**), Gli2 (**D**), Gli3 (**E**), and smoothened (SMO) (**F**). Relative band intensity normalized to that of β-actin. Ihh, Runx2, Sox9, Gli2, Gli3, and SMO increased significantly after 12-hour cyclic tensile strain in cells derived from patients with OPLL (OPLL cells, n = 8). There was no significant increase in each factors from patients without OPLL (non-OPLL cells, n = 6). *p < 0.05.

To confirm the expression of genes identified using microarrays, Ihh and related signaling factors were evaluated by immunoblot analysis. Changes in levels of Ihh, Runx2, Sox9, SMO, Gli2, and Gli3 after cyclic tensile strain in cultured OPLL cells and non-OPLL cells in immunoblot analysis are shown in Fig. 2. Under a non-stress condition, the expression levels of Ihh, Sox9, and SMO were significantly higher in OPLL cells than in non-OPLL cells. The expression levels for Ihh, Runx2, Sox9, Gli2, and Gli3 in OPLL cells increased significantly after 6-hour cyclic tensile strain. SMO increased significantly after 6-hour cyclic tensile strain, but decreased after 24 hours.

### Histopathological and Immunohistochemical staining

**Figure 3:**
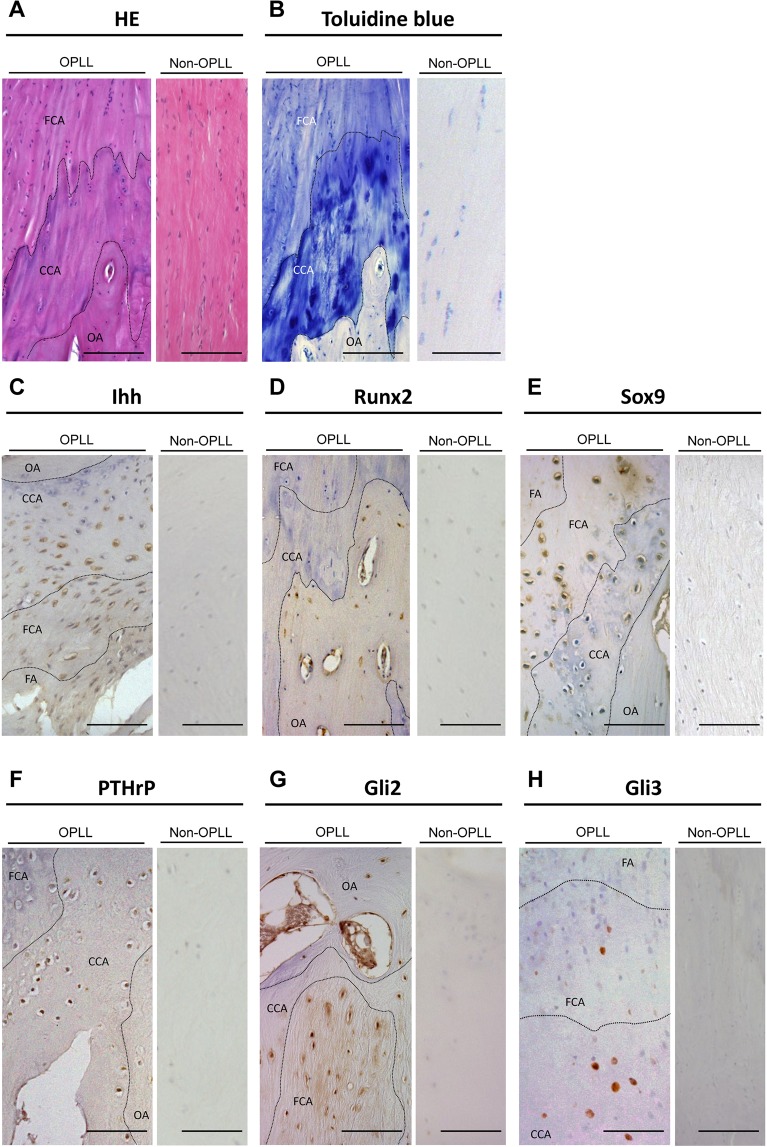
(**A,B**) A section of OPLL had an expanding ossification front with abundant chondrocytes around the calcification front. A section of the non-OPLL posterior longitudinal ligament showing a regular arrangement of fiber bundles without a chondrometaplastic area. (**A**) H&E; (**B**) Toluidine blue. **(C–H**) Immunohistochemical staining of ossified posterior longitudinal ligament (OPLL) tissue. (**C**) Ihh is strongly expressed in proliferating chondrocytes. (**D**) Runx2 is expressed in proliferating chondrocytes in the fibrocartilage area. (**E**) Sox9 is strongly expressed in proliferating chondrocytes. (**F**) PTHrP is expressed in hypertrophic chondrocytes. (**G**) Gli2 is expressed in proliferating chondrocytes and cartilage matrix around the hypertrophic chondrocytes. (**H**) Gli3 is expressed in hypertrophic chondrocytes. Non-OPLL tissue are negative for each factor. Scale bar =100 μm. OA, ossified area; CCA, calcified cartilage area; FCA, fibrocartilage area.

To confirm the expression of genes identified using microarrays, Ihh and related signaling factors were evaluated by immunohistochemical analysis. In patients with OPLL, the ossification front had features with an irregular arrangement. The calcified cartilage layer and calcification front extended longitudinally to the fibrocartilage area. Around the calcification front, hypertrophic chondrocyte-like cells were present. In contrast, this layered structure was not observed in patients with non-OPLL, although a regular structure with a few chondrocytes was present (Fig. 3A,B). Table 4 summarized the results of immunohistochemical analysis. In OPLL tissue, Ihh was strongly expressed in chondrocytes, particularly in proliferating chondrocytes in the fibrocartilage area near the calcification front (Fig. 3C). Proliferating chondrocytes in the fibrocartilage area had positive immunoreactivity for Runx2 (Fig. 3D). Sox9 was expressed in proliferating chondrocytes, but not in hypertrophic chondrocytes (Fig. 3E). PTHrP was expressed around the calcification front in the calcified cartilage area (Fig. 3F). Gli2 was expressed in proliferating chondrocytes and cartilage matrix around the hypertrophic chondrocytes (Fig. 3G), and Gli3 was expressed in hypertrophic chondrocytes in the calcified cartilage area (Fig. 3H). In contrast, Ihh, Sox9, Runx2, PTHrP, Gli2, and Gli3 were not expressed in non-OPLL tissue.

**Table 4:** Summary of immunohistochemical staining of the ossification front.

	Ossified area (OA)	Hypertrophic chondrocytes (CCA)	Proliferating chondrocytes (FCA)	Fiber area (FA)
Ihh	±	+	++	−
Runx2	−	−	+	±
Sox9	±	−	++	±
PTHrP	−	+	±	−
Gli2	±	+	+	−
Gli3	−	+	±	−

++, strongly positive; +, moderately positive; ±, weakly positive; −, negative staining

OA, ossified area; CCA, calcified cartilage area; FCA, fibrocartilage area, FA, fiber area.

## Discussion

OPLL is a multifactorial disease that involves genetic and environmental factors^21^. Among the environmental factors, cyclic tensile strain has an important local effect on ossification in OPLL^22^. Cyclic tensile strain is an important regulator of bone metabolism, and increases both the number of osteoblasts and the expression levels of various osteogenic genes, such as alkaline phosphatase (ALP), type I collagen, osteocalcin, and osteopontin^23^. In a previous study, we showed that enlargement of the nucleus pulposus followed by herniation, disruption, and regenerative proliferation of cartilage in the annulus fibrous participated in initiation of calcification of the PLL in *twy/twy* mice. In addition, after cells of the protruded hyperplastic annulus fibrosus invaded the longitudinal ligaments, these cells induced metaplasia of primitive mesenchymal cells to osteoblasts with neovascularization^24^.

Regarding genetic effects, there is a high prevalence of OPLL in certain countries and races, and several reports have identified underlying genetic factors. The previous study demonstrated that patients with OPLL had a significantly higher incidence of genetic abnormalities in the type XI collagen (alpha) 2 gene (COL11A2) region^25^, and genome-wide linkage study showed that a single-nucleotide polymorphism in the collagen 6A1 gene (COL6A1) was strongly associated with OPLL^26^. In a DNA microarray analysis, angiopoietin-1 levels in cultured OPLL cells were significantly higher after adding chondrogenic factors, such as connective tissue growth factors or cartilage oligomeric matrix protein^27^. Another study identified six susceptibility gene loci for progression of OPLL at 20p12.3, 8q23.1, 12p11.22, 12p12.2, 8q23.3, and 6p21.1 in a genome-wide association study^28^. In addition, we have found that expression levels for Ihh, PTHrP, and Sox9 are significantly higher in cultured cells from patients with OPLL than in cells from non-OPLL patients^14^. In our study, ALP activities of cultured cells derived from OPLL patients were significant higher than the cells from non-OPLL patients. These findings indicated that cultured OPLL cells have both osteogenic and cartilaginous (osteochondral bi-potential) characters.

Cyclic tensile strain also influences upregulation of expression of genes involved in progression of OPLL. A previous study demonstrated that mechanical stress due to cyclic stretching led to upregulation of several genes associated with bone metabolism, including endothelin (ET)-1 and prostaglandin I_2_, in OPLL cells^29^. Spinal ligament cells derived from patients with OPLL had osteoblastic phenotypes and higher sensitivity to mechanical stress, compared with spinal ligament cells from patients without OPLL^30,31^. In the present study, many osteogenic and chondrogenic markers, including Ihh signaling, were upregulated by cyclic tensile strain in a microarray analysis although there were individual differences in the results of gene expression. These results suggest that expression of Ihh and its related signaling factors might be sensitive to cyclic tensile strain, and additional local cyclic tensile strain might induce abnormal changes in expression of these genes.

We reported previously that OLF cells activate their ossification properties under cyclic tensile strain, via the Wnt/β-catenin signaling pathway^18^. In addition, the other study reported that mRNA levels for ALP, osteopontin, BMP2, BMP4, and BMP receptors were significantly increased by cyclic stretching in cultured cells derived from the ligamentum flavum of patients with OPLL^17^. In the present study, the expression levels for receptors of Ihh, such as Gli2 and Gli3, increased significantly after 6-hour cyclic tensile strain in immunoblotting analysis. Interestingly, Gli2 and Gli3 were upregulated before Ihh, Son9, Runx2, and SMO were upregulated. In patients with OPLL, the PLL is subjected to strain stress for a long time, and ossification progresses very slowly; however, our results demonstrate that cyclic tensile strain might affect the receptors first, followed by upregulation of Ihh, Sox9, Runx2, and SMO by strain stress.

In normal enchondral ossification, Ihh, a member of the Hedgehog family, is an essential factor for chondrocyte differentiation. In the growth plate, Ihh is expressed in prehypertrophic chondrocytes and causes the surrounding tissue to differentiate into metaphasic tissues^32–34^. Ihh with Sox9 or PTHrP induces differentiation and maturation of chondrocytes in the early stage of normal enchondral ossification, and Ihh induces calcification with Runx2 in the late stage by controlling maturation of chondrocytes^35,36^. In this study, Ihh was expressed in proliferating chondrocytes in the ossification front of human cervical OPLL plaques. Therefore, we speculate that Ihh might be involved in the ossification process in human OPLL, as in normal enchondral ossification.

Previous immunohistochemical studies have shown that chondrocytes near the ossification front express transcriptional factors and collagen types that are involved in ossification. The previous study described that BMP2 contributed to cartilage and bone formation and that TGF-β stimulated bone formation in the ossified ligaments of patients with OPLL^37^. We previously reported that type I collagen was significantly expressed in the matrix of the ossified PLL, whereas type II collagen was highly expressed in metaplastic chondrocytes at the ossification front, and that apoptotic hypertrophic chondrocytes were observed near the calcification front^14^. In the present study, we showed that Ihh, Runx2 and Sox9 are expressed in proliferating chondrocytes. Furthermore, we demonstrated expression of PTHrP, Gli2 and Gli3 in hypertrophic chondrocytes. The presence of Gli2, Gli3, and SMO in the ossification front provides evidence that Ihh and its related signaling factors might contribute to the ossification process in human OPLL.

In conclusion, Gli2 and Gli3 were strongly upregulated after 6-hour cyclic tensile strain followed by upregulation of Ihh, Sox9, Runx2, and SMO, and genes related to chondrogenesis, including Ihh, Sox9, and Runx2, were upregulated after 24-hour strain in cultured OPLL cells. In addition, Ihh, Runx2, and Sox9 proteins were expressed in the proliferating chondrocyte layer, PTHrP, Gli2 and Gli3were expressed in the hypertrophic chondrocyte layer at the ossification front of OPLL. These results suggest that cyclic tensile strain might induce upregulation of genes and proteins related to differentiation or maturation of chondrocytes, and that Ihh and its related signaling factors might play a key role in human OPLL.

The limitation of this study was that there were differences in results between the *in vivo* and *in vitro* studies using human OPLL-related samples. Thus, cellular responses in cultured cells cannot be extrapolated with certainty to the *in vivo* situation of cells located in the extracellular matrix. Also, the cyclic tensile strain used for cultured cells was applied over a shorter period to that *in vivo*. Thus, the effects of mechanical stress on expression of signal transduction factors in OPLL requires further study.

## Methods

### Patients and biological samples

*En bloc* samples of the ligament-ossified plaque complex were obtained from 49 patients (29 men, 20 women; mean age 69.6 years) who underwent anterior decompressive surgery at our hospital. OPLL was diagnosed based on the presence of bone formation in the PLL on radiography or computed tomography of the cervical spine. None of the patients had evidence of congenital bone or joint disorders or musculoligamentous tissue abnormalities, and none had seronegative spondyloarthropathy or hyperparathyroidism or was being treated with glucocorticoids or immunosuppressants. The surgical procedures for cervical anterior decompression and interbody fusion are described in detail in our previous publication^11^. Briefly, a left-sided anterolateral approach was used to expose the affected vertebral levels. The OPLL plaque was isolated circumferentially, together with the surrounding non-ossified PLL (deep layer), like a floating “island” on the dura mater. Using a custom-made micro-Kerrison rongeur, the non-ossified area of the ligament peripheral to the ossified lesion was carefully dissected and removed *en bloc* with the surrounding tissues. Samples were also obtained from 7 patients without OPLL (cervical disc herniation: CDH) who underwent cervical anterior decompressive surgery (4 men, 3 women; mean age 63.4 years) for use as age-matched control materials (Table 1). Before surgery, written informed consent was obtained from each patient. The study protocol was approved by the Human Ethics Review Committee of our University Medical Faculty and strictly followed the Clinical Research Guidelines of the Ministry of Health, Labor, and Welfare of the Japanese Government.

### Histopathological staining

Resected OPLL plaque together with the surrounding posterior longitudinal ligaments tissue was bisected mid-sagittally, followed by fixation with 10% buffered formaldehyde for 48 hours at 4 °C. The sample was further decalcified for 4 to 7 days at 4 °C in 0.5 M EDTA and 0.5 M Tris-HCl buffer at pH 7.6, and then embedded in paraffin using standard procedures. Serial 4-µm thick sagittal sections of the OPLL were prepared for hematoxylin and eosin (H&E) and Toluidine-Blue staining.

### Cell culture

For cell culture, PLL specimens were harvested from 9 of the 49 patients with OPLL (7 men, 2 women; mean age at surgery 68.4 years; range 59–79 years: OPLL cells). Samples were also obtained from 7 patients without OPLL who underwent cervical anterior decompressive surgery (4 men, 3 women; mean age 63.4 years; range 56–75 years: non-OPLL cells) for use as age-matched control materials (Table 1). Cultured PLL cells derived from the vicinity of the OPLL plaque (OPLL cells) were isolated as described previously^14,18^. Briefly, the ligaments were harvested aseptically from the ossified tissue, minced into approximately 0.5-mm^3^ pieces, and explanted onto 100-mm diameter dishes in 10 ml of Dulbecco’s Modified Eagle Medium (#12320, Low Glucose 1X, Lot 561521, GIBCO, Grand Island, NY) supplemented with 10% qualified fetal bovine serum (#1395965, GIBCO) and 1% penicillin/streptomycin (#15140, 667553, GIBCO) and incubated at 37 °C in a humidified atmosphere of 95% air/5% CO_2_. The cultures were left undisturbed for 48 h, followed by replacement of the medium with an equal volume of fresh medium. Cells derived from the explants were harvested from the dishes with 0.02% ethylenediaminetetraacetic acid and 0.05% trypsin for further passages, and used in experiments after the third passage.

### ALP activity

Alkaline phosphatase activity was evaluated using an ALP stain kit (Takara Bio Inc., Shiga, Japan). For each sample of non-stressed cultured non-OPLL and OPLL cells, a micrograph of 10 fields was obtained for analysis, and the ratio of positively stained cells to total cells was determined.

### ***In vitro*application of cyclic tensile strain to cultured cells**

A Flexercell^®^ FX-3000 cell stretching device (Flexercell International, Hillsborough, NC) was used in this study. The device consists of a computer-controlled vacuum unit and a culture-well plate with a flexible-polystyrene culture bottom coated with type I collagen (Flex I, Bioflex^®^ Plates, Flexercell^®^ International). The culture plates are six-well (35-mm diameter) flexible bottomed culture plates with a hydrophilic surface. Application of a vacuum provides a hemispherically downward deformation force on the flexible bottom, resulting in a non-homogenous strain profile with a maximum at the periphery and a geometric decline toward zero at the center of the culture well bottom. Based on a previous study in which the same device was used, we set the tensile strain at a maximum of 20% elongation^18,27^. Selection of this strain level was also based on the other previous studies. Panjabi and White showed that the elastic properties of the human spinal ligament are markedly altered when they are subjected to 26% elongation^20^. The other study assessed 0–30% elongation and selected 20% elongation at 0.5 Hz because expression of ALP was reported to increase with an increasing extension ratio to reach a plateau at an extension ratio of 30%, and cell damage occurred once the extension ratio exceeded 30%^27^. To show the validity of this strain condition using in this study, cell morphology and viability were examined, as described below. For this experiment, OPLL cells were subjected to cycles of 10 s of 20% elongation and 10 s of relaxation for 6, 12, or 24 h. For microarray analysis, three OPLL cell lines were prepared without strain and with 24-hour strain, and two non-OPLL cell line was used as a control. For immunoblotting analysis, eight OPLL cell lines and six non-OPLL cell lines were prepared without strain and with 6-, 12-, and 24-hour strain.

### Quantification of cell survival

Cell viability of cultured ligament cells was examined using a Live/Dead assay (Cambridge Biosciences, Cambridge, UK) under a confocal microscope (Leica TCS SP2, Wetzlar, Germany). Ethidium homodimer-1 cannot penetrate live cells but stains the DNA of dead cells red. Calcein-AM penetrates live cells, in which an esterase cleaves the molecule, which then fluoresces green, whereas dead cells do not contain the esterase. For visualization, the cells were soaked in the Live/Dead solution for 1 hour. The numbers of live (green) and dead (red) cells were counted manually. The cell survival rate was calculated.

### Microarray analysis

For microarray analysis, 3 OPLL and 2 non-OPLL cells were used (Table 1). After cyclic tensile stretching, the cultured cells were disrupted in a lysis buffer containing β-mercaptoethanol, and total RNA extracts from the cells were pooled at each time point and further purified using RNeasy^®^ (Qiagen, Valencia, CA). A quality check of each total RNA sample was carried out using an Agilent 2100 Bioanalyzer (Agilent, Palo Alto, CA). Total RNA (100 µg) was then reverse-transcribed into double-stranded cDNA using oligo-dT primers. Biotinylated cRNA was synthesized using an *in vitro* transcription labeling kit (Agilent). Following fragmentation, 20 μg of cRNA was hybridized for 17 h at 65 °C on a Agilent SurePrint G3 Human GE 8x60K v2 Microarray. Gene chips were washed and scanned using an Agilent DNA Microarray Scanner (Agilent). The scanned images were quantified using Agilent Feature Extraction 7 software (Agilent), and the raw data for each hybridization were calculated. The obtained microarray data were initially processed using Gene Spring GX 12.0 software (Agilent). The ratio and differences in intensity between two corresponding genes on each array were calculated. A significant difference in the levels of gene expression was defined as a ratio of the difference of intensity of >2.0.

GO and KEGG analysis were performed for categorizing identified genes, and identifying specific genes and pathways by enrichment analysis. The Gene Ontology Consortium (www.geneontology.org/doc/GO.doc.html) maintains a controlled vocabulary database of functional descriptions for genes. In our study, we used the total list of GO terms within the biological process categories. The KEGG system (http://www.genome.jp/kegg/) provides a reference knowledge base for linking genomes to life through the process of pathway mapping. A list of genes in OPLL cultured cells with at least a 2.0-fold change in expression and a p-value <0.05 compared with those in non-OPLL cells were used for KEGG pathway analyses.

### Immunoblot analysis

For immunoblot analysis, 8 OPLL and 6 non-OPLL cells were used (Table 1). An immunoblot analysis method previously described by our laboratory was used to evaluate expression levels of transcriptional factors^11,14,18^. Briefly, the sample was solubilized in RIPA buffer, homogenized, and then stored at −80 °C. The protein concentration was determined using a Bio-Rad DC protein assay kit (Lot 500–0116, Bio-Rad Laboratories, Hercules, CA). Total protein (80 µg/lane) was subjected to sodium dodecylsulfate polyacrylamide gel (15%) electrophoresis (SDS-PAGE) and transferred onto a polyvinylidene difluoride membrane in a semi-dry blot apparatus. The membranes were then washed twice in PBS and subsequently reacted with primary antibodies overnight at 4 °C and then with an anti-rabbit IgG antibody and avidin-biotinylated peroxidase complex (1:200; Envision™ System-HRP Labeled Polymer, Dako) for 3 h. The primary antibodies used in this study were rabbit polyclonal anti-Ihh (lot 20450) (GeneTex, San Antonio, TX, USA), rabbit polyclonal anti-Sox9 (lot D406), rabbit polyclonal anti-Runx2 (lot G1009) (both Santa Cruz Biotechnology, Santa Cruz, CA, USA), rabbit polyclonal anti-Gli2 (lot 584061) (CosmoBio, Tokyo, Japan), rabbit polyclonal anti-Gli3 (Lot GR79855–4), and rabbit polyclonal anti-smoothened (SMO: lot GR198520–1) (both Abcam, Tokyo, Japan). After triple washing in 0.1 M PBS, the membrane was immersed and subjected to radiography to visualize the peroxidase activity and thus the level of antibody binding. To quantify the relative level of expression of the four evaluated transcriptional factors in cultured OPLL and non-OPLL cells, the bands on the photographic film were analyzed with a densitometer using NIH image analysis software (ver. 1.59/ppc, NIH, Bethesda, MD). Data are expressed semi-quantitatively as the ratio of the density of each band to that of β-actin (1:500, rabbit IgG; Abcam).

### Immunohistochemical staining

For immunohistochemical staining, PLL specimens were harvested from 40 of the 49 patients with OPLL (23 men, 17 women; mean age at surgery 69.9 years). Samples were also obtained from 3 patients without OPLL who underwent cervical anterior decompressive surgery (1 man, 2 women; mean age 60.7 years) for use as age-matched control materials (Table 1). Tissue blocks were stained immunohistochemically, as described previously^11,14^. The primary antibodies used in this study (sources given above, unless stated) were rabbit polyclonal anti-Ihh, rabbit polyclonal anti-Sox9, rabbit polyclonal anti-Runx2, mouse monoclonal anti-PTHrP (batch 17011; AbD Serotec, Oxford, UK), rabbit polyclonal anti-Gli2, and rabbit polyclonal anti-Gli3. Signals were detected by reaction with goat anti-mouse immunoglobulin antibodies conjugated to a peroxidase labeled-dextran polymer (EnVision^TM^, Peroxidase, Dako, Glostrup, Denmark) for monoclonal antibodies and with goat anti-rabbit immunoglobulin antibodies (EnVision + , Peroxidase, Dako) for polyclonal antibodies at 20 °C for 45 min, followed by rinsing with PBS at pH 7.4. To visualize the peroxidase color reaction, sections were incubated with DAB-HCl solution (CB090; Dojin Chemicals, Tokyo, 50 mg dissolved in 100 ml of 0.05 M Tris-HCl buffer at pH 7.4) at 20 °C for 10 min. Nuclear counterstaining was carried out with hematoxylin. Semiquantitative analysis conducted according to the method previously described^11,14,18,38^. Briefly, we indicated the degree of immunoreactivity as: −not labeled or tissue was negative; ±, weak labeling above background or only traces of the tissue were stained; +, moderate labeling above background or less than one-third of the cells were stained; ++, intense labeling above background or more than one-third of the cells were stained.

### Statistical analysis

All values are expressed as mean ± SD. Differences between groups were tested by one-way analysis of variance (ANOVA), with *p* < 0.05 in a Tukey post hoc analysis considered to indicate significance. For measurement of stained tissues, the inter- and intraobserver reliabilities were assessed by calculating intraclass correlation coefficients (ICC), with ICC values < ±0.75–1.00 considered to show excellent reliability. All statistical tests were performed using SPSS ver. 24.0 (SPSS, Chicago, IL).

## Supplementary information

Supplementary Table S1.

## Data Availability

Data generated and analyzed during this study are included in this published article. Data and materials are available from the corresponding author subject to reasonable request and subject to the ethical approvals in place and materials transfer agreements.
