# Supplementary material for: Cyclic tensile strain facilitates ossification of the cervical posterior longitudinal ligament via increased Indian hedgehog signaling
Source: Sci Rep. 2020 Apr 29;10:7231. doi: 10.1038/s41598-020-64304-w (PMC7190672; doi:10.1038/s41598-020-64304-w)
Supplement: Supplementary file 1 — Supplementary Table S1. [file 41598_2020_64304_MOESM1_ESM.docx]

**Cyclic tensile strain facilitates ossification of the cervical posterior longitudinal ligament via increased Indian hedgehog signaling**

Daisuke Sugita, MD, PhD, Hideaki Nakajima, MD, PhD*, Yasuo Kokubo, MD, PhD, Naoto Takeura, MD, Takafumi Yayama, MD, PhD, Akihiko Matsumine, MD, PhD.

Table S1. Top 30 GO terms enrichment results of up-regulated genes in OPLL cultured cells with expression change > 2.0-fold compared to those in non-OPLL cultured cells

| **Rank** | **GO term** | **Count** | **p value** | **Fold Enrichment** |
| --- | --- | --- | --- | --- |
| **After 24-hour cyclic tensile strain** | | | | |
| 1 | type I interferon signaling pathway | 18 | 1.3E-10 | 7.46 |
| 2 | defense response to virus | 24 | 6.0E-8 | 3.86 |
| 3 | response to virus | 17 | 3.5E-6 | 4.10 |
| 4 | extracellular matrix organization | 23 | 4.9E-6 | 3.11 |
| 5 | negative regulation of viral genome replication | 10 | 0.000014 | 6.63 |
| 6 | angiogenesis | 23 | 0.000037 | 2.74 |
| 7 | positive regulation of endothelial cell proliferation | 12 | 0.000049 | 4.61 |
| 8 | cell adhesion | 36 | 0.000063 | 2.08 |
| 9 | positive regulation of endothelial cell migration | 9 | 0.00028 | 5.19 |
| 10 | interferon-gamma-mediated signaling pathway | 11 | 0.00031 | 4.11 |
| 11 | positive regulation of ERK1 and ERK2 cascade | 18 | 0.00033 | 2.73 |
| 12 | positive regulation of macrophage chemotaxis | 5 | 0.00053 | 12.1 |
| 13 | positive regulation of cell migration | 18 | 0.00060 | 2.60 |
| 14 | leukocyte migration | 14 | 0.00069 | 3.04 |
| 15 | neutrophil chemotaxis | 10 | 0.00078 | 4.02 |
| 16 | positive regulation of GTPase activity | 38 | 0.00079 | 1.78 |
| 17 | integrin-mediated signaling pathway | 12 | 0.0012 | 3.22 |
| 18 | negative regulation of protein kinase activity | 12 | 0.0012 | 3.22 |
| 19 | protein kinase B signaling | 7 | 0.0013 | 5.63 |
| 20 | positive regulation of angiogenesis | 13 | 0.0013 | 3.00 |
| 21 | positive regulation of smooth muscle cell proliferation | 9 | 0.0018 | 3.98 |
| 22 | inflammatory response | 27 | 0.0024 | 1.89 |
| 23 | activation of protein kinase B activity | 6 | 0.0025 | 6.12 |
| 24 | positive regulation of phosphatidylinositol 3-kinase signaling | 9 | 0.0029 | 3.67 |
| 25 | ossification | 10 | 0.0031 | 3.32 |
| 26 | embryonic skeletal system morphogenesis | 7 | 0.0031 | 4.76 |
| 27 | positive regulation of MAPK cascade | 10 | 0.0033 | 3.28 |
| 28 | regulation of chondrocyte differentiation | 4 | 0.0038 | 11.8 |
| 29 | cellular response to extracellular stimulus | 5 | 0.0040 | 7.37 |
| 30 | apoptotic signaling pathway | 9 | 0.0050 | 3.36 |
|  |  |  |  |  |
| **Under non-stress condition** | | | | |
| 1 | nucleosome assembly | 21 | 4.9E-08 | 4.41 |
| 2 | type I interferon signaling pathway | 14 | 1.2E-6 | 5.47 |
| 3 | negative regulation of transcription from RNA polymerase II promoter | 56 | 3.1E-6 | 1.94 |
| 4 | defense response to virus | 19 | 0.00011 | 2.88 |
| 5 | extracellular matrix organization | 21 | 0.00012 | 2.68 |
| 6 | negative regulation of cell growth | 15 | 0.00034 | 3.10 |
| 7 | innate immune response in mucosa | 7 | 0.00037 | 7.00 |
| 8 | response to virus | 14 | 0.00044 | 3.18 |
| 9 | negative regulation of viral genome replication | 8 | 0.00093 | 5.00 |
| 10 | integrin-mediated signaling pathway | 12 | 0.0020 | 3.03 |
| 11 | peripheral nervous system development | 6 | 0.0023 | 6.25 |
| 12 | positive regulation of smooth muscle cell proliferation | 9 | 0.0025 | 3.75 |
| 13 | response to drug | 24 | 0.0026 | 1.97 |
| 14 | cell-matrix adhesion | 11 | 0.0031 | 3.05 |
| 15 | phosphatidylinositol-mediated signaling | 12 | 0.0034 | 2.83 |
| 16 | cellular response to extracellular stimulus | 5 | 0.0049 | 6.94 |
| 17 | intracellular signal transduction | 28 | 0.0063 | 1.74 |
| 18 | defense response to Gram-positive bacterium | 10 | 0.0068 | 2.94 |
| 19 | regulation of heart contraction | 6 | 0.0072 | 4.84 |
| 20 | antibacterial humoral response | 7 | 0.0077 | 3.98 |
| 21 | negative regulation of axon extension | 4 | 0.0082 | 9.09 |
| 22 | chromatin silencing | 7 | 0.0086 | 3.89 |
| 23 | negative regulation of gene expression | 13 | 0.0088 | 2.37 |
| 24 | response to mechanical stimulus | 8 | 0.0089 | 3.39 |
| 25 | cellular heat acclimation | 3 | 0.0091 | 18.7 |
| 26 | positive regulation of transcription, DNA-templated | 33 | 0.0093 | 1.60 |
| 27 | heterotypic cell-cell adhesion | 5 | 0.010 | 5.68 |
| 28 | negative regulation of cyclin-dependent protein serine/threonine kinase activity | 5 | 0.010 | 5.68 |
| 29 | inositol phosphate metabolic process | 7 | 0.011 | 3.72 |
| 30 | protein O-linked fucosylation | 4 | 0.011 | 8.33 |
